# Mediastinal gray zone lymphoma in a pregnant woman presenting with cardiac tamponade

**DOI:** 10.1186/s40959-023-00173-2

**Published:** 2023-05-31

**Authors:** Azin Alizadehasl, Kamran Roudini, Mahshid Hesami, Farid kosari, Hamid Reza Pouraliakbar, Mina Mohseni, Negar Dokhani

**Affiliations:** 1 Cardio-Oncology Research Center, Rajaie Cardiovascular Medical and Research Center, Iran University of Medical Sciences, Tehran, Iran; 2 Department of Internal Medicine, Hematology and Medical Oncology Ward, Cancer Research Center, Cancer Institute, Imam Khomeini Hospital Complex, Tehran University of Medical Sciences, Tehran, Iran; 3 Rajaie Cardiovascular Medical and Research Center, Iran University of Medical Sciences, Tehran, Iran; 4 Department of Pathology, School of Medicine Shariati Hospital, Tehran University of Medical Sciences, Tehran, Iran; 5 Department of Radiology, Rajaie Cardiovascular, Medical and Research Center, Iran University of Medical Sciences, Tehran, Iran

**Keywords:** Gray zone lymphoma, Pericardial effusion, Cardiac tamponade, Pregnancy, Chemotherapy

## Abstract

**Background:**

Mediastinal gray zone lymphoma is a newly recognized rare B cell neoplasm, which is challenging in diagnosis and treatment.

**Case presentation:**

In the current study, we aimed to report a 25-year-old pregnant woman at 25 weeks of gestation who presented with chronic cough and progressive shortness of breath, hypotension, tachycardia, and tachypnea. A large circumferential pericardial effusion with compressive effect on the right atrium and right ventricle and a large extracardiac mass with external pressure to mediastinal structures were seen on trans thoracic echocardiography. The emergency pericardiocentesis was performed with the diagnosis of cardiac tamponade. Also, CMR revealed a huge heterogeneous anterior mediastinal mass, and the pathology and the immunohistochemistry of the mass biopsy revealed gray zone lymphoma with positive CD3, CD20, CD30, CD45, PAX5, and negative CD15 expression. Three courses of chemotherapy with the CHOP regimen were performed with an acceptable response every three weeks before delivery. A caesarian section was performed at 37 weeks without any problem for the patient and fetus, and chemotherapy will be started three weeks after delivery.

**Conclusion:**

Cardiac tamponade as an emergency condition occurred in this pregnant patient by malignant pericardial effusion and mediastinal mass pressure. Accurate diagnosis and on time interventions caused a significant improvement and a successful delivery.

## Background

Mediastinal gray zone lymphoma was described as a “missing link” with intermediate features between the classic Hodgkin's lymphoma and mediastinal large B cell lymphoma in 2005. Overlap in immunophenotype, histopathology, and clinical features complicates definitive diagnosis and treatment [1]. Lymphoma is the fourth most frequent malignancy in pregnancy, occurring in 1 out of 6000 deliveries. Limitations in the usage of imaging modalities to staging the tumor and choosing the appropriate treatment options during pregnancy have always been a challenge for physicians [2]. Here we introduce a pregnant patient presenting to the hospital with cardiac tamponade caused by an anterior mediastinal mass with morphologic and IHC staining of gray zone lymphoma.

## Case presentation

A 25-year-old primigravida female was admitted at 25 weeks of gestation with severe dyspnea that had progressed over the last month. Additionaly, she noticed a progressive, productive cough that started six months ago. At presentation, she had a regular pulse rate of 140 beats per minute, a Blood Pressure of 90/50 mmHg, and a respiratory rate of 30 breaths/min. O_2_ saturation was 94% in the room air, and she was afebrile. Cardiac examinations revealed muffled heart sounds, pulsus paradoxus, and elevated jugular venous pressure. Other examinations and her past medical history were unremarkable, and she denied experiencing any constitutional sign. Her electrocardiogram showed sinus tachycardia. On echocardiography, the size and the systolic function of the right and left ventricles were normal. There was evidence of a large pericardial effusion with significant right atrial invagination and RVOT diastolic collapse. There were significant respiratory variations of TV and MV inflow velocities, and IVC appeared plethoric. A large extra-cardiac mass at the pulmonary valve site adjacent to distal RVOT was seen, resulting in turbulency in pulmonic outflow without significant gradient and stenosis.

The blood tests showed neutrophilic leukocytosis with a white blood cell count of 16,410 cells/mm3, a hemoglobin level of 9.2 g/dl, a platelet count of 403*103 /mm3, an ESR level of 95 mm/h, an LDH level of 300 IU/L, and CRP more than 90 with standard coagulation test. The results of the pericardial fluid analysis are presented in the Table 1.

**Table 1:** Pericardial Fluid analysis

Pericardial Fluid analysis	
Pericardial Fluid Glucose	107 ml/dl
Pericardial Fluid Protein	7 g/dl
Pericardial Fluid LDH	4380 IU/L
Pericardial Fluid Alkaline Phosphatase	128 U/L
Pericardial Fluid Cholesterol	89 ml/dl
Pericardial Fluid Triglyceride	44 ml/dl
Pericardial Fluid RBC	0.3⨯10^6^ cells/µL
Pericardial Fluid WBC	2499 cells/µL (Neut:75% Lymph:25%)
ADA fluid	22
Pericardial Fluid culture	Negative

**Fig. 1:**
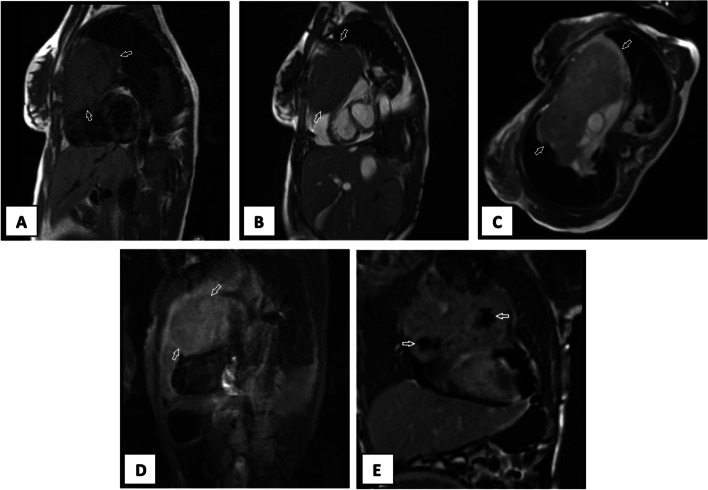
Cardiac magnetic resonance **A** Short axis T1-W sequence view shows iso-signal tumor. **B**, **C** Short axis and axial SSFP sequence views reveal heterogeneous high signal tumor. **D** Short axis STIR sequence view demonstrates high signal tumor. **E** Coronal Late Gadolinium Enhancement (LGE) shows heterogeneous enhancement and necrosis (arrow). SSFP = steady-state free precession, STIR = Short tau inversion recovery)

Cardiac magnetic resonance determined a 146*126*136^ mm^ heterogeneous mass in the anterior mediastinum attached to the pericardium with a compressive effect on RVOT Fig. 1.

**Fig. 2:**
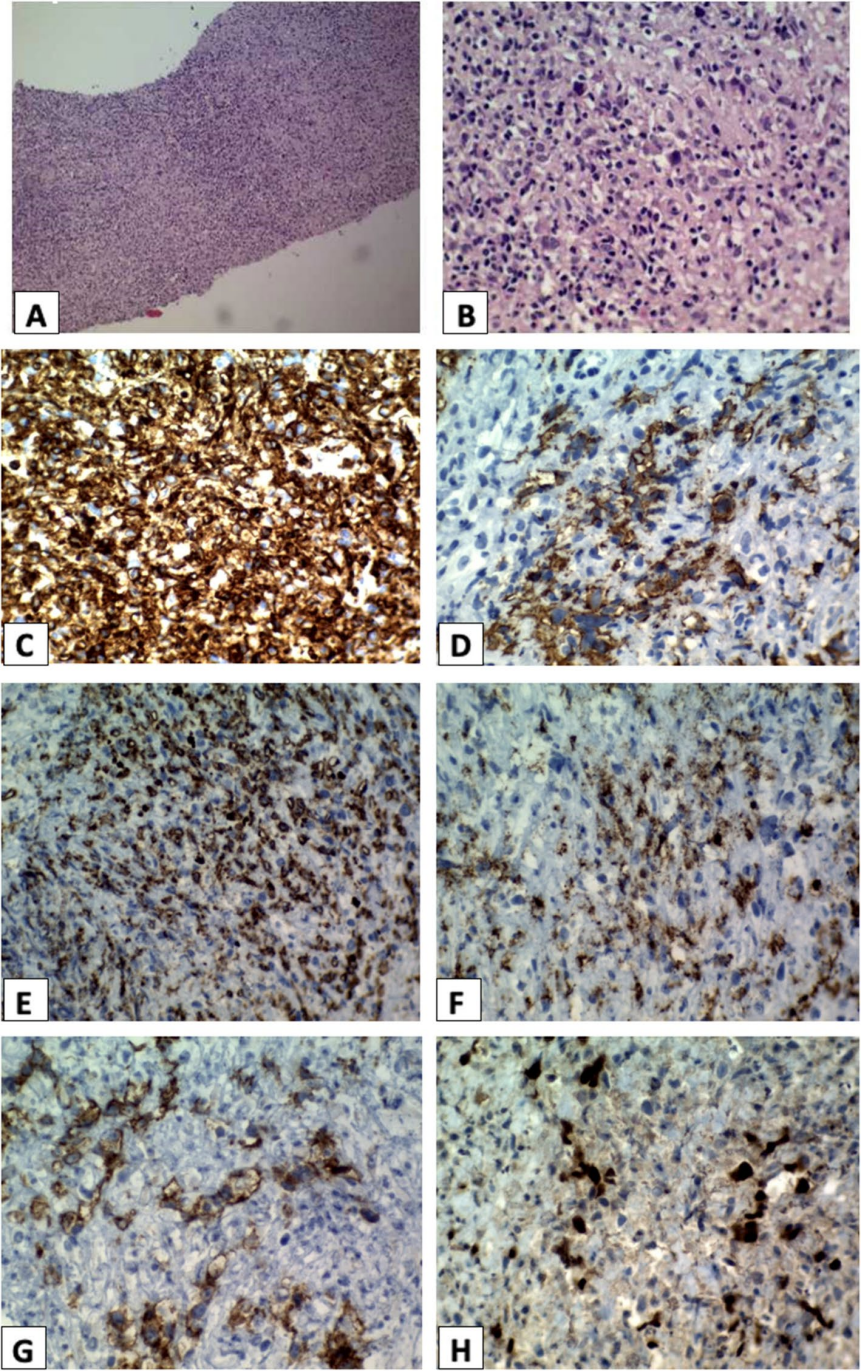
Mediastinal mass pathology **A**, **B** Microscopic examination (H&E staining) shows diffuse and nodular infiltration of lymphocytes, neutrophils, eosinophils and some large atypical cells with enlarged hyperchromatic nuclei with irregular contour and prominent nucleoli in fibrotic stroma. **C** All lymphoid cells including some large atypical cells are positive for CD45. **D** Many large atypical cells are positive for CD20. **E** Many small lymphocytes are positive for CD3. **F** Large atypical cells are negative for CD15. **G** Large atypical cells are positive for CD30. **H** Large atypical cells are strongly positive for PAX5)

A mass biopsy was performed, and the pathology revealed diffuse and nodular infiltration of lymphocytes, neutrophils, eosinophils, and atypical cells with enlarged hyperchromatic nuclei with irregular contours and prominent nucleoli in the fibrotic stroma. The immunohistochemistry showed negative CD15 and positive CD3, CD20, CD30, CD45, PAX5 expression. According to the clinical and paraclinical findings, the diagnosis of gray zone lymphoma was confirmed Fig. 2.

Subcutaneous Pericardiocentesis under fluoroscopy with a subxiphoid approach was performed, and after drainage of 200CC serous fluid, the pigtail catheter was fixed under negative pressure. While the patient's hemodynamic status improved, it did not fully recover until receiving the high dose of dexamethasone.

An oncology consultation was requested, and dexamethasone was started at 40 mg daily for four days. Three courses of chemotherapy were administered using the CHOP regimen (Cyclophosphamide 750/m^2^, Hydroxydaunorubicin 50/m^2^, Vincristine Sulfate 2 mg, and Prednisone 75 mg/m^2^ day1-day5) with intervals of twenty-one days. The treatment was stopped three weeks before delivery to avoid the coincidence of the next date of chemotherapy with delivery and prevent any potential complications for the mother and fetus, such as bleeding during childbirth and neonatal myelosuppression [3]. The patient demonstrated an improvement in the symptoms that prompted her admission, indicating a suitable response to chemotherapy. All the fetal ultrasounds performed during chemotherapy were normal. After completing two courses of chemotherapy and giving birth, the mother underwent trans-thoracic echocardiography to monitor her cardiac health. The result showed normal LV and RV function, as well as normal LVEF, with no presence of pericardial effusion.

An elective cesarean section was performed at 37 weeks of gestation. The mother's health status during and after delivery was good, and her son was in perfect health with no observable abnormalities. Chemotherapy will commence three weeks after delivery.

## Discussion

Cardiac involvement, which can occur as a secondary complication of malignancies or as a result of chemotherapy agents or radiation therapy, represents a significant challenge in cancer treatment. The pericardium can be affected in the form of pericarditis or pericardial effusion by local invasion, obstruction of lymphatic and venous drainage, and hematogenous or lymphatic spread during malignancies. Lung, breast, leukemia, and lymphoma are the most common neoplasms with malignant pericardial effusion [4]. Cardiac tamponade is a life-threatening medical emergency that requires immediate intervention. The compression of heart chambers through tamponade is caused by fluid, gas, or extracardiac mass effect and leads to decreased cardiac output and shock [5].

The term "Gray zone lymphoma" was first used to delineate the border cases between classic Hodgkin's and non-Hodgkin's lymphoma in 1998; then, in 2008, included in the WHO classification of lymphoid neoplasm as an "unclassifiable B-cell lymphoma, with features intermediate between diffuse large B-cell lymphoma and classical Hodgkin lymphoma" and finally in 2022 WHO named this entity as Mediastinal Gray Zone lymphoma (MGZL) [6–8]. Overlap in immunophenotype, histopathology, and clinical features complicates definitive diagnosis and treatment. CD45, CD20, PAX5, BOB1, CD79a, OCT2 expression, and the absence of CD15 expression are common immunohistochemical findings in MGZL. Strong expression of CD20 and PAX5 as B cell associated markers, frequently not seen in CHL. CD45 expression is highly specific for DLBCL and reported in 100% of non-Hodgkin's lymphoma. CD15 expression is positive in 75–90% of CHL cases, and CD30 is more expressed in CHL than DLBCL. Differences in therapeutic regimen and lower survival rate compared to CHL and DLBCL demonstrate the importance of identifying MGZL to find an appropriate treatment for this neoplasm [9].

Physiologic changes in pregnancy can imitate or hide the cancer symptoms and lead to a delay in diagnosis. Cancer staging and treatment are also challenging in pregnancy because of the risk that threatens the mother and fetus [10]. Currently, the R-CHOP regimen is the prefered treatment for gray zone lymphoma [11]. chemotherapy without Rituximab was started for the patient because the evidence showed CHOP regimen is safe beyond the first trimester [12]. It is recommended to schedule the delivery of pregnant cancer patients between 37 and 39 weeks of gestation, with vaginal delivery being the optimal mode of childbirth [13]. Although the preferred mode of delivery was explained to the patient, she refused to consider a vaginal birth. She insisted on cesarean birth, which was ultimately performed at 37 weeks and six days of gestation.

## Conclusions

Cardiac tamponade is an emergency condition that needs immediate pericardiocentesis. Tamponade occurred in this patient by mediastinal mass pressure and malignant pericardial effusion. The simultaneous occurrence of cardiac tamponade and mediastinal gray zone lymphoma, a rare neoplasm in pregnancy, creates a complex condition for patient and physicians.

## Data Availability

The authors can confirm that all relevant data are included in the article.

## Abbreviations

RVOT: Right ventricular outflow tract
TV: Tricuspid valve
MV: Mitral valve
IVC: Inferior vena cava
CMR: Cardiac magnetic resonance
R-CHOP: Rituximab + (Cyclophosphamide, Hydroxydaunorubicin, vincristine sulfate, and Prednisone)
CHL: Classic Hodgkin’s lymphoma
DLBCL: Diffuse large B cell lymphoma
